# Elucidating the Mechanism of Large Phosphate Molecule Intercalation Through Graphene-Substrate Heterointerfaces

**DOI:** 10.1021/acsami.3c07763

**Published:** 2023-10-02

**Authors:** Jiayun Liang, Ke Ma, Xiao Zhao, Guanyu Lu, Jake Riffle, Carmen M. Andrei, Chengye Dong, Turker Furkan, Siavash Rajabpour, Rajiv Ramanujam Prabhakar, Joshua A. Robinson, Vasquez Magdaleno, Quang Thang Trinh, Joel W. Ager, Miquel Salmeron, Shaul Aloni, Joshua D. Caldwell, Shawna Hollen, Hans A. Bechtel, Nabil D. Bassim, Matthew P. Sherburne, Zakaria Y. Al Balushi

**Affiliations:** † Department of Materials Science and Engineering, University of California, Berkeley, Berkeley, California 94720, United States; ‡ Materials Sciences Division, Lawrence Berkeley National Laboratory, Berkeley, California 94720, United States; § Department of Mechanical Engineering, Vanderbilt University, Nashville, Tennessee 37235, United States; ∥ Department of Physics and Astronomy, University of New Hampshire, Durham, New Hampshire 03824, United States; ⊥ Canadian Centre for Electron Microscopy, McMaster University, Hamilton ,ON L8S 4L8, Canada; # 2D Crystal Consortium, The Pennsylvania State University, University Park, Pennsylvania 16802, United States; ∇ Department of Materials Science and Engineering, The Pennsylvania State University, University Park, Pennsylvania 16802, United States; ○ Chemical Sciences Division, Lawrence Berkeley National Laboratory, Berkeley, California 94720, United States; ◆ Department of Mining, Metallurgy, and Materials Engineering, University of the Philippines, Diliman, Quezon City 1101, Philippines; ¶ Queensland Micro- and Nanotechnology Centre, Griffith University, Brisbane, 4111 Australia; †† The Molecular Foundry, Lawrence Berkeley National Laboratory, Berkeley, California 94720, United States; ‡‡ Advanced Light Source, Lawrence Berkeley National Laboratory, Berkeley, California 94720, United States; §§ Department of Materials Science and Engineering, McMaster University, Hamilton ,ON L8S 4L8, Canada

**Keywords:** graphene, intercalation, heterointerface, reactions, defects

## Abstract

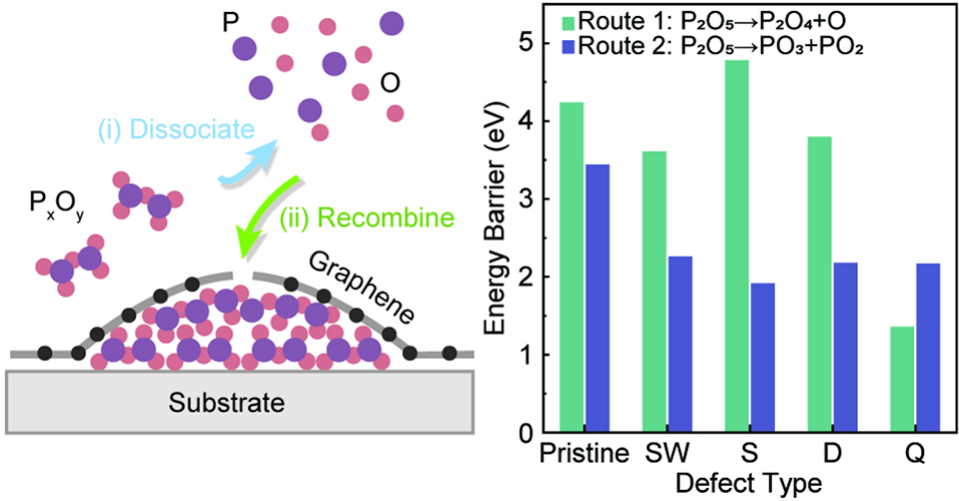

Intercalation is the process of inserting chemical species into the heterointerfaces of two-dimensional (2D) layered materials. While much research has focused on the intercalation of metals and small gas molecules into graphene, the intercalation of larger molecules through the basal plane of graphene remains challenging. In this work, we present a new mechanism for intercalating large molecules through monolayer graphene to form confined oxide materials at the graphene-substrate heterointerface. We investigate the intercalation of phosphorus pentoxide (P_2_O_5_) molecules directly from the vapor phase and confirm the formation of confined P_2_O_5_ at the graphene-substrate heterointerface using various techniques. Density functional theory (DFT) corroborates the experimental results and reveals the intercalation mechanism, whereby P_2_O_5_ dissociates into small fragments catalyzed by defects in the graphene that then permeates through lattice defects and reacts at the heterointerface to form P_2_O_5_. This process can also be used to form new confined metal phosphates (e.g., 2D InPO_4_). While the focus of this study is on P_2_O_5_ intercalation, the possibility of intercalation from predissociated molecules catalyzed by defects in graphene may exist for other types of molecules as well. This in-depth study advances our understanding of intercalation routes of large molecules via the basal plane of graphene as well as heterointerface chemical reactions leading to the formation of distinctive confined complex oxide compounds.

## Introduction

Intercalation is a topotactic insertion process of organic or inorganic chemical species (i.e., atoms, small molecules, etc.) between the interfaces and heterointerfaces of two-dimensional (2D) layered materials. This process is known to form a variety of intercalation compounds, notably in graphite, encompassing a diverse range of intercalants within the host of the 2D layered bulk crystal.^1^ Intercalation can occur through the exposed side edges of a 2D layered bulk crystal and/or through its basal planes.^2^ In the latter case, for example, in monolayer to few-layer graphene, the intercalation pathways are typically point defects,^3−9^ and/or grain boundaries.^10^ Usually, the intercalation process can occur using a variety of processes, including vapor transport,^11^ wet-chemical,^12−15^ and electrochemical^16^ means. To date, much of the research surrounding the intercalation of monolayer graphene has focused on the intercalation of metals (e.g., Ga, In, Al,^17^ Bi,^18^ Fe,^19^ Sb,^20^ Gd,^21^ Pb,^22^ etc.) and small gas molecules (e.g., CO,^23^ O_2_^24^). However, the intercalation of molecules through the basal plane of monolayer graphene that are significantly larger than the lattice parameters of graphene remain challenging. This is because the size and energy barrier for intercalation inhibits the permeation of such large molecules.^25,26^ The ability to intercalate large molecules could further expand the toolsets for subsurface and heterointerface engineering of 2D layered materials and therefore enrich the material choices available for the fabrication of heterostructures and intercalation compounds used in energy storage, optoelectronics, thermoelectric, catalysis, etc.^27−29^

Chemical reactions enabled by confinement to the heterointerfaces of 2D layered materials provide a pathway to circumvent the limitation on intercalating large molecules directly through the basal plane. That is, individual chemical species or multiple small fragments of the initial state of the molecule would first intercalate through typically available pathways in the graphene lattice, and then recombine at the heterointerface via chemical reactions. So far, a variety of alloys (Sn_1–*x*_Ge_*x*_,^30^ Fe_1–*x*_Co_*x*_,^31^ etc.) and compounds (GaN,^32−34^ AlN,^35^ MoS_2_,^36^ PtSe_2_,^37^ Ga_2_O_3_,^38^ etc.) have been formed at graphene-substrate heterointerfaces via chemical reactions. In all of these cases, however, chemical species are individually introduced in a sequential manner when intercalating graphene. This is done to prevent prereactions in the vapor phase between the individual elements prior to intercalation. Such prereactions usually lead to the formation of clusters at defect sites on the graphene surface rather than intercalating through the lattice itself and forming a compound at the heterointerface.

In this work, we report a mechanism of vapor phase intercalation of large molecules through the heterointerface of monolayer graphene to form confined oxide materials underneath graphene with a chemical composition that resembles the initial state of the large intercalant molecule itself. Unlike prior work, where chemical species and/or elements were sequentially introduced into the heterointerface, single molecules were introduced directly into the vapor phase for intercalation through the basal plane. This mechanism was revealed by investigating the intercalation of phosphorus pentoxide (P_2_O_5_) molecules directly from the vapor phase to form P_2_O_5_ at a graphene-substrate heterointerface. The formation of confined P_2_O_5_ was confirmed using a variety of chemical analysis and spectroscopic techniques as well as microscopic surface and cross-sectional imaging. Density functional theory (DFT) was used to corroborate experimental results and to deduce the mechanism for the intercalation of P_2_O_5_ molecules through the basal plane of monolayer graphene. First, P_2_O_5_ dissociates into small fragments (i.e., P_*x*_, O_*y*_, etc.) when interacting with the graphene surface. These small fragments consistently showed lower energy barriers for intercalation through defects commonly observed in the graphene lattice. Once the small fragments intercalate, they react at the heterointerface to form P_2_O_5_. We show that the intercalation of P_2_O_5_ at the graphene-substrate heterointerface can tune the electronic structure of the graphene overlayer through strain and charge transfer. In addition, we show that this process can also be used in the formation of new confined metal phosphates by intercalating P_2_O_5_ into a graphene-substrate heterointerface initially containing a confined 2D indium metal to form InPO_4_. Although this study focuses on the intercalation of P_2_O_5_, the concept of intercalation from predissociated molecules catalyzed by defects in graphene could be possible in other classes of molecules. Ultimately, this study provides an important steppingstone for advancing the understanding of intercalation pathways of large molecules though the basal plane of graphene as well as heterointerface chemical reactions to form unique confined complex oxide compounds.

## Results and Discussion

**Figure 1:**
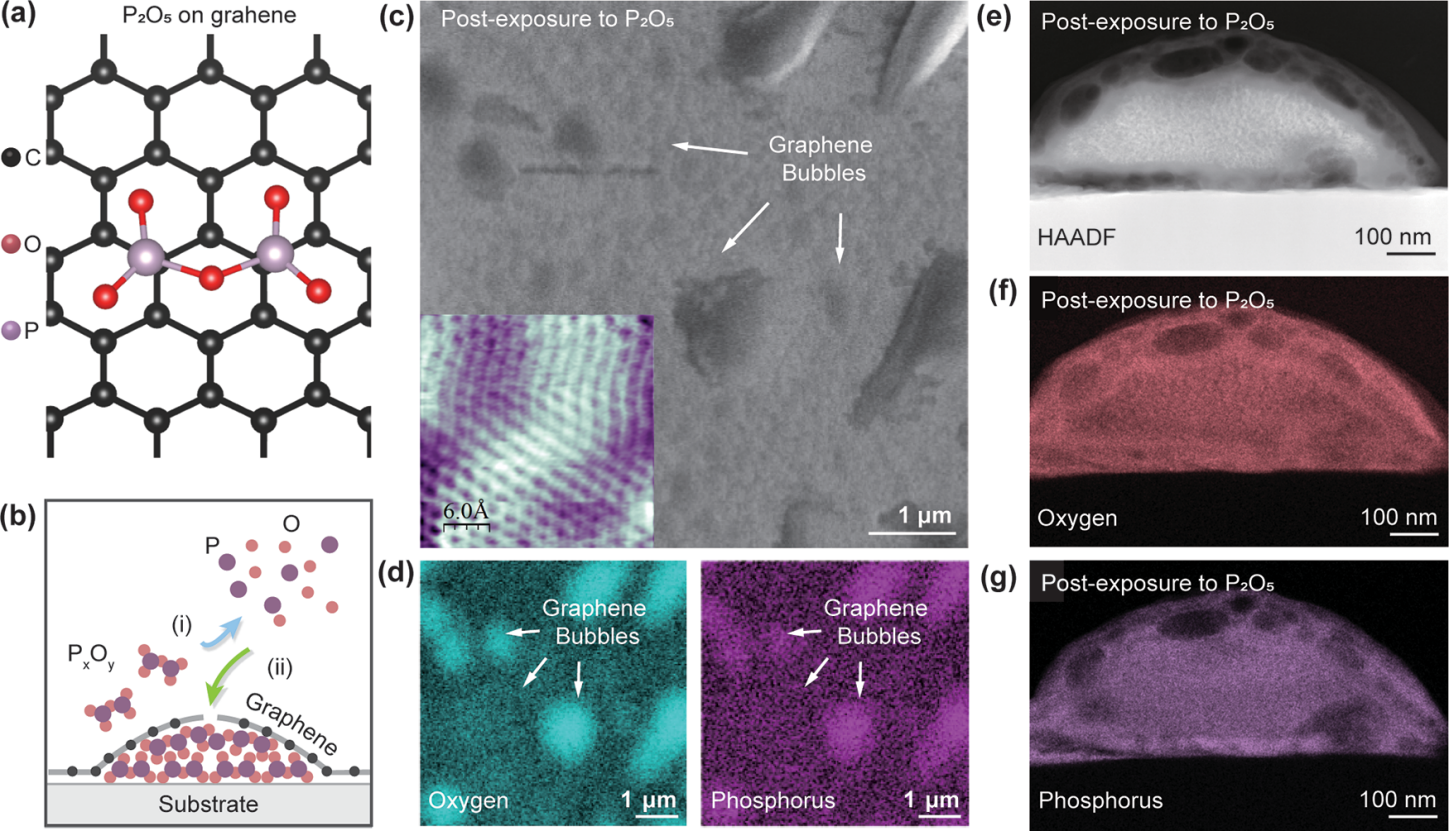
Intercalation of P_2_O_5_ at the heterointerface of graphene-germanium. (a) Configuration of P_2_O_5_ adsorbed onto pristine graphene calculated from DFT. (b) Schematic of intercalation process through the graphene-substrate heterointerface: (i) decomposition of P_2_O_5_ into P_*x*_ and O_*y*_, (ii) intercalation of P_*x*_ and O_*y*_ through graphene and formation of P_2_O_5_ at heterointerface via chemical reactions. (c) SEM highlighting the formation of graphene bubbles (white arrows), with an STM image of the graphene lattice on the top surface of the bubble (bottom left inset). (d) SEM-EDS maps of (left) oxygen and (right) phosphorus of the highlighted graphene bubbles in panel (c). (e) STEM-HAADF cross-section image of a graphene bubble with corresponding STEM-EDS maps of the (f) oxygen and (g) phosphorus distribution in the bubble.

The physical size of phosphorus pentoxide (P_2_O_5_) can be deduced from our DFT calculated ground state structure when it is adsorbed onto the surface of pristine monolayer graphene (Figure 1a). This molecule consists of two main phosphorus bonds with oxygen. The P–O single bond (denotated as oxygen bridging) has a bond length of 157–159 pm, and the P=O double bond (denotated as oxygen terminal) has a bond length of 143.2–144.5 pm.^39^ When the physical size of the molecule is intuitively compared to the lattice parameter of graphene, intercalation through the basal plane appears to be unlikely. Our DFT calculations revealed that the intercalation of P_2_O_5_ molecules in its native state through graphene could only occur if large pores, formed by the removal of 9 carbon atoms, were present (see the Supporting Information). The formation of such large pores in graphene is, however, energetically unfavorable.^40^ In fact, other theoretical studies have predicted large energy barriers for permeation of many atoms and molecules through pristine graphene.^25,26^ In our case, we experimentally show that it is possible to intercalate P_2_O_5_ through monolayer graphene transferred onto germanium substrates without the need for large pores to serve as intercalation pathways. In our studies, germanium was used as a substrate due to the limited solubility^41^ and low diffusivity^42^ of phosphorus into germanium, allowing to fully capture and study the formation of P_2_O_5_ at graphene-germanium heterointerfaces. To intercalate P_2_O_5_, we prepared monolayer graphene on germanium substrates which were then annealed downstream of SiP_2_O_7_ at 950°*C* (see Methods). At such temperatures, the decomposition of SiP_2_O_7_ produced an upstream flux where P_2_O_5_ is the predominant gas-phase specie, as supported by previously reported experimental and computational studies.^43−45^ P_2_O_5_ then impinges onto the surface of graphene downstream and subsequently intercalates into the heterointerface (Figure 1b). A more detailed discussion of the mechanism is included in the following sections.

As shown in Figure 1c, many bubbles appeared on the surface of graphene after samples were exposed to a flux of P_2_O_5_ (white arrows in scanning electron micrograph, SEM). From scanning tunneling microscopy (STM) imaging of the bubble surface (Figure 1c, inset), the characteristic hexagonal lattice of graphene was clear. This suggests that not only was the graphene surface free of large pores but also highlighted that the bubbles themselves were “graphene bubbles” containing subsurface material. Such graphene bubbles are prevalent in many intercalation studies of monolayer to few-layer graphene in the literature.^25,46^ Upon further inspection of the surface using energy-dispersive spectroscopy (EDS), strong phosphorus and oxygen signals confined to the graphene bubbles were observed (Figure 1d). This implied the formation of phosphorus oxides within the bubbles themselves. The distribution of the phosphorus and oxygen in the graphene bubbles was further corroborated in sample cross sections. In Figure 1e, a representative high-angle annular dark field scanning transmission electron microscope (HAADF-STEM) image of a graphene bubble is shown. The EDS cross-sectional elemental maps of the bubble (Figure 1f,g) also revealed strong oxygen and phosphorus signals. The atomic fractions of oxygen and phosphorus in the graphene bubble were 55.06% and 15.52%, respectively. This elemental distribution was further corroborated within X-ray photoelectron spectroscopy (XPS) depth profiles of the sample, facilitated by repeated ion sputtering (Figure S1). During the ion sputtering process, the graphene surface was first etched. This resulted in a rapid decrease in the C 1s core-level peak intensity (Figure S1a). The peak intensities for the Ge, O, and P core levels increased upon sputtering of graphene (Figure S1b–d), which serves as additional evidence on the confinement of phosphorus oxide at the graphene–germanium substrate heterointerface after samples were exposed to a flux of P_2_O_5_.

### Chemistry of the Graphene–Substrate Heterointerface

**Figure 2:**
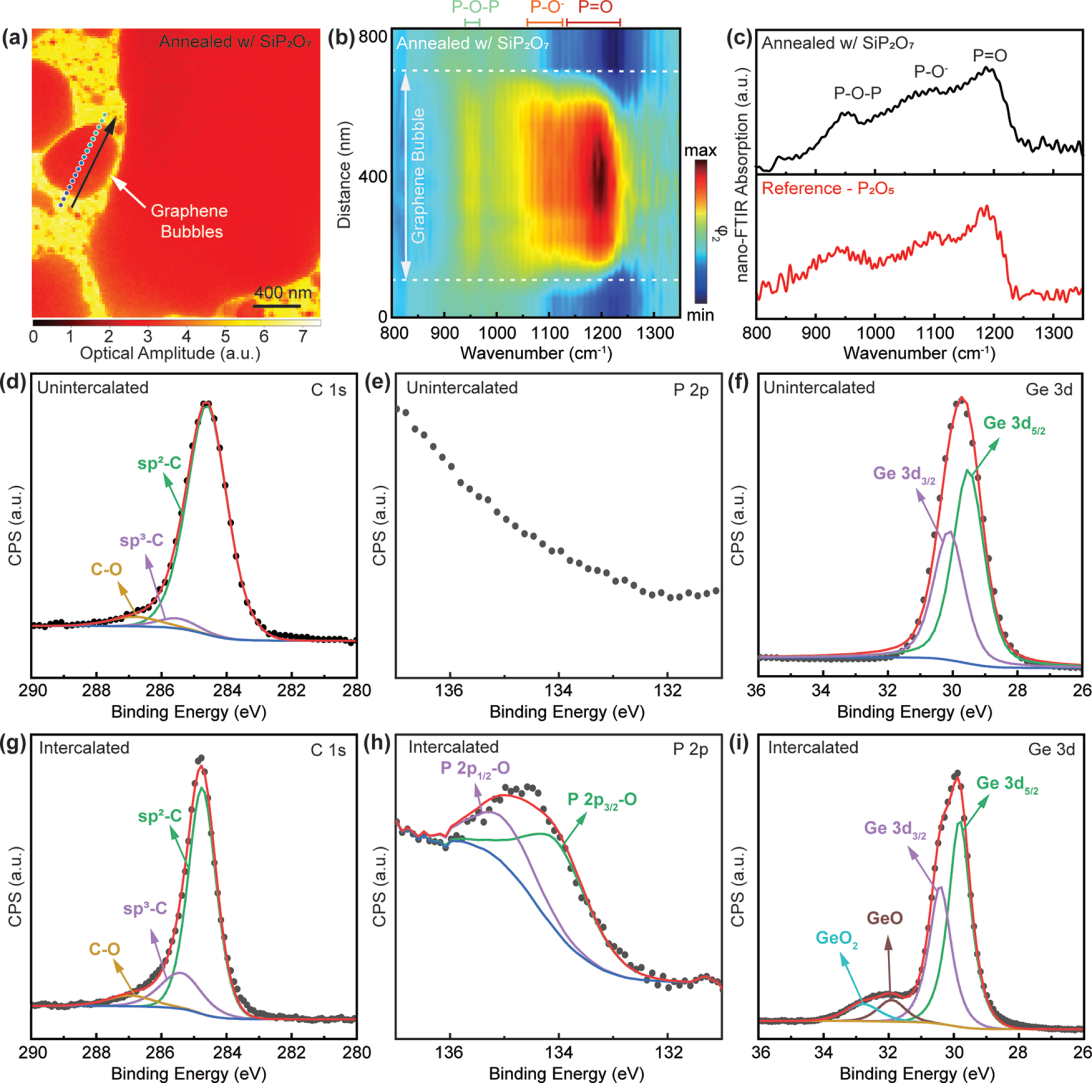
Chemistry at the graphene-germanium heterointerface. (a) Near-field white light IR image in a region of the sample containing large graphene bubbles. The white light IR image is the near-field amplitude (*S*_2_) image, which is spectrally averaged over approximately ∼610–1400 cm^–1^ by setting the interferometer at the white light position. (b) Nano-FTIR spectral heat map acquired at 50 nm intervals along the black solid arrow in panel (a). The graphene bubble region is highlighted with white dashed lines and a dashed arrow. The collected phase channel (φ_2_) was normalized to that obtained in a silicon sample using the same acquisition parameters. (c) Comparison between extracted nano-FTIR spectrum of a graphene bubble (top panel, black solid line) and spectrum collected from a reference P_2_O_5_ sample (bottom panel, red solid line). XPS of C, P, and Ge core levels from graphene on germanium samples (d–f) annealed without an upstream flux of P_2_O_5_ (i.e., unintercalated sample) and from graphene on germanium samples (g–i) annealed with an upstream flux of P_2_O_5_ (i.e., intercalated sample).

Moreover, the chemical environment of phosphorus oxide within the graphene bubbles was investigated using nano Fourier-transform infrared spectroscopy (nano-FTIR). A near-field white light IR image of a region of the sample containing graphene bubbles, spectrally averaged over ∼610–1400 cm^–1^, is highlighted in Figure 2a. Spectral points in the range associated with vibrational modes associated with the phosphorus and oxygen functional groups were acquired at 50 nm intervals across a graphene bubble (black arrow). This is plotted in Figure 2b as a near-field phase (φ_2_) heat map, which is related to the local absorption spectra of the material, and therefore, useful for determining the chemical environment of the intercalant.^47,48^Figure 2c highlights a comparison between the two nano-FTIR spectra. The top panel (black solid line) is the extracted phase spectrum at the center of the graphene bubble in Figure 2b, while the bottom panel (red solid line) is a spectrum collected from a reference sample of P_2_O_5_ using high-resolution synchrotron-based nano-FTIR measurements (see Methods). Evident in Figure 2b and the top panel of Figure 2c are three characteristic IR bands localized to the graphene bubble. These bands were assigned as the P–O–P bending mode (940–970 cm^–1^), the P–O bridging mode (1060–1125 cm^–1^), and the P=O terminal oxygen stretching modes (1135–1235 cm^–1^).^49^ The intensity of the P=O bond in the heat map (Figure 2b) reached a maximum at the center of the graphene bubble. More importantly, these peaks well matched the P_2_O_5_ reference sample in Figure 2c (bottom panel) and therefore supports phosphorus oxide that was confined at the graphene–germanium substrate heterointerface from the cross-section micrograph in Figure 1g was P_2_O_5_.

Furthermore, a comparative XPS study between nonintercalated and intercalated samples was also performed to further deduce the chemical bonding information at the graphene-germanium heterointerface. Figure 2d–f are XPS core-level spectra collected from samples that were annealed without an upstream flux of P_2_O_5_, while the XPS core levels in Figure 2g–i were from samples annealed downstream to a flux of P_2_O_5_ (see Methods). In the nonintercalated samples, the sp^3^/sp^2^ carbon ratio, extracted from the C 1s core level, was ∼4% (Figure 2d), and neither phosphorus oxides (Figure 2e) nor germanium oxides (Figure 2f) could be detected in those samples. However, in intercalated samples, the sp^3^/sp^2^ carbon ratio increased to ∼7.7% (Figure 2g). Such an increase in sp^3^-bonded carbon within the nonintercalated samples was likely due to the formation of additional point defects in the graphene lattice. These defects could have resulted from the intercalation process itself or due to the enhanced hybridization of the graphene with the underlying intercalants. Moreover, a strong phosphorus oxide peak, whose binding energy was associated with P_2_O_5_ (135 eV, Figure 2h), further corroborated its formation.^50^ Also, from the Ge 3d core-level, the intercalated P_2_O_5_ appeared to oxidize the germanium substrate, resulting in characteristic peaks for GeO and GeO_2_ that were observed between binding energies of 31–33 eV (Figure 2i). These combined results allude to a strong affinity of oxygen from P_2_O_5_ to the underlying germanium substrate and perhaps provides an additional driving force in the intercalation process with the aid of point defect and/or grain boundaries in the graphene lattice as a pathway for intercalation through the basal plane of graphene.^8^

### Mechanism of Large Molecule Intercalation through Graphene

**Figure 3:**
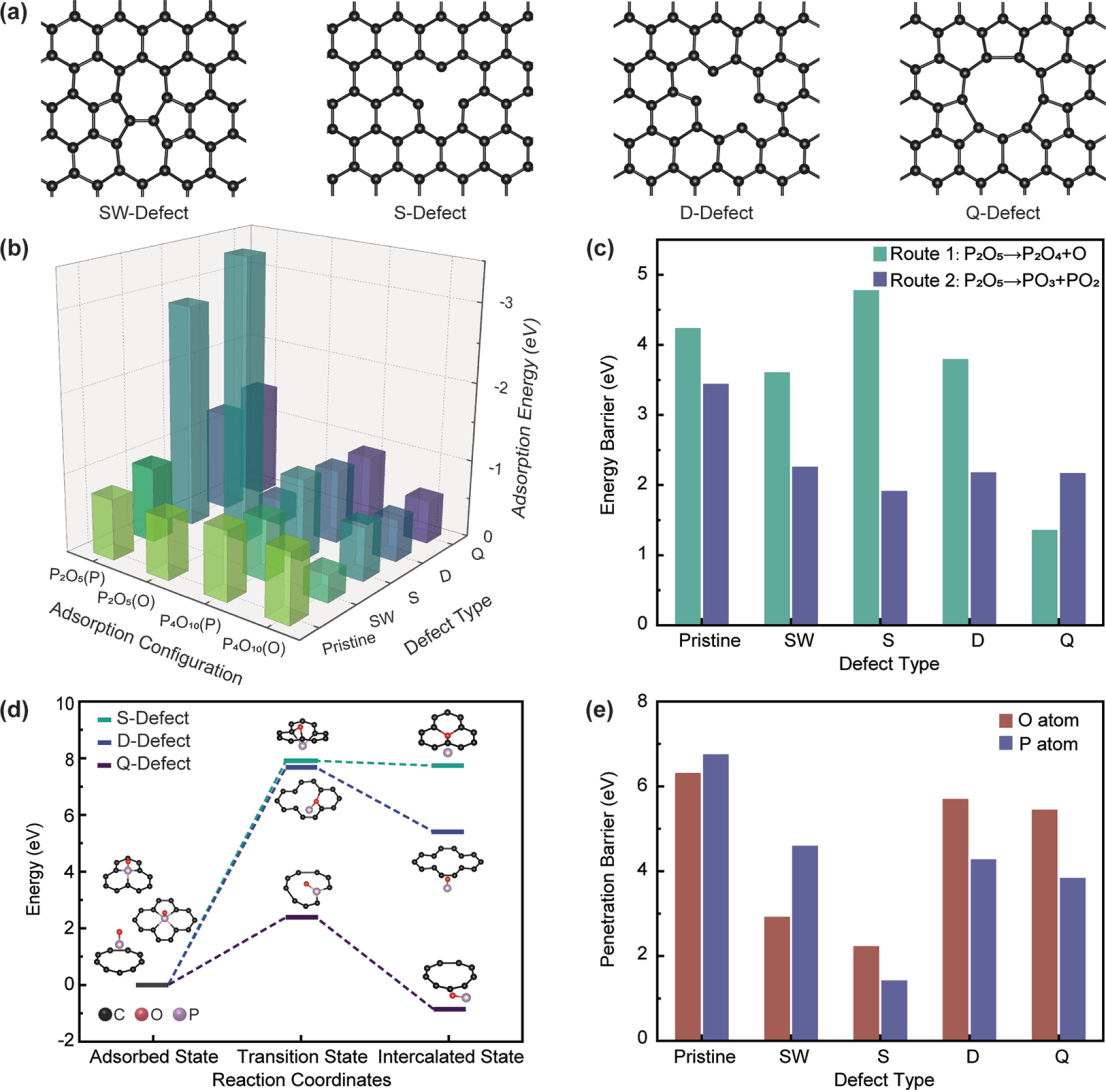
Mechanism for intercalation P_2_O_5_ through the basal plane of graphene. (a) Top view of the four types of point defects in isolated graphene sheets. (b) Adsorption energy of phosphorus pentoxide on pristine and defective graphene monolayers. Within each P_*x*_O_*y*_ molecule, the atom interacting directly with carbon at the defect site is labeled in parentheses. (c) Calculated energy barrier of bond cleavage for P=O double bond (route 1, labeled as green) and single bond (route 2, labeled as blue) within P_2_O_5_ in different local environments. (d) Energy and configurational profile of PO molecule penetrating through defects. (e) Energy barrier of P (blue) and O (red) atoms permeating through defects.

To gain a deeper understanding into the mechanism by which phosphorus pentoxide P_2_O_5_ (or molecular formula P_4_O_10_) intercalates through the basal plane of graphene, we first examined the adsorptive properties of these molecules on five different graphene systems, consisting of pristine graphene and four graphene lattice defect configurations (Figure 3a, see the Supporting Information). This survey was performed to factor in different interaction parameters of distinct chemical environments on the intercalation process. The configurational space of the adsorptive states (chemisorption vs physisorption) was thoroughly explored by including a multitude of initial interacting geometries for both P_2_O_5_ and P_4_O_10_ on each of the different graphene lattice defect configurations (Figure 3b). As summarized in Table S1, covalent interactions with graphene occurred exclusively in the chemisorption of P_2_O_5_ onto graphene defect sites, whereas in pristine graphene, P_2_O_5_ interacted weakly via van der Waals (vdW) forces. In comparison, interactions between the more stable P_4_O_10_ molecule and all graphene systems were vdW in nature. In the case of covalent bonding between P_2_O_5_ adsorbents and graphene, P atoms consistently demonstrated a higher affinity toward graphene. This is shown by the greater adsorption energy of P_2_O_5_ to graphene through the P atom (denoted by P_2_O_5_(P) in Figure 3b). In contrast, initial configurations of P_2_O_5_ approaching graphene with its O atom (designated by P_2_O_5_(O)) either relaxed to drastically different interaction geometries to allow bond formation between carbon and phosphorus or had a much lower total adsorption energy when compared to P_2_O_5_(P). The only exception was P_2_O_5_(O) chemisorbed to S defects, in which both the atoms of the O and P bonded covalently to the graphene defect site. Such trends can be rationalized by the fulfillment of covalency and increased charge transfer stabilization of carbon atoms at defects when bonding to less electronegative P atoms in P_2_O_5_.

Pristine graphene has been predicted to be impermeable to most gaseous species under nonextreme conditions,^25,26,46^ and the presence of defects is thus the precondition for P_2_O_5_ intercalation. A quantitative description of intercalation through defects is complicated by the competition between diffusion and bonding with carbon atoms at defect sites. This gives rise to three different energy profiles corresponding to distinct pathways.^51^ To address these complexities, we employed a case-by-case strategy by calculating the penetration barriers for fragments of P_2_O_5_ of varied size and chemistry based on calculated and experimental evidence from the literature.^45^ Based on experimental evidence of P_2_O_5_ confinement at the graphene-germanium heterointerface, the intercalation of molecular P_2_O_5_ in its entirety was first investigated. Point defects of various sizes were examined, with the largest created by removing 9 adjacent carbon atoms in an isolated graphene sheet (Figure S2). In all scenarios, P_2_O_5_ decomposed into fragments solvated by carbon atoms at the defect sites. Notably, the energetic profile of P_2_O_5_ permeating through large defects showed a negative reaction coordinate energy curve with small intermediate energy barriers as a result of the bonding of the fragmented P_2_O_5_ molecule to the defect site. Despite this negative reaction coordinate energy curve, a barrier of at least 7.7 eV was still present for fragments to desorb from the defect and diffuse into opposite sides of the monolayer graphene (i.e., intercalate). From this, we ascertain that intercalation is only possible from significantly smaller molecular fragments of P_2_O_5_. Developed from these findings, we propose a mechanism involving three consecutive steps of realistic energy barriers to intercalate P_2_O_5_ through the basal plane of monolayer graphene. First, chemisorbed P_2_O_5_ decomposes into atoms or small molecular fragments. The resulting species then intercalate separately through point defects and then react underneath the graphene monolayer to form P_2_O_5_ at the confined heterointerface.

Describing such processes with a full network of reaction pathways would require a dedicated theoretical effort and is beyond the scope of this study, but two modes of P–O bond cleavage representing the initial steps of two probable pathways were investigated to exemplify our hypothesis. As illustrated in Figure 3c, route 1 involves cleaving the P=O double bond to remove one terminal oxygen from the molecule, while route 2 describes the fragmentation of P_2_O_5_ into PO_2_ and PO_3_ by cleaving the single bond between P and bridging O. Energy barriers for both reactions are shown in Figure 3c. Evidently, with the exception of S-defect in route 1, all four types of point defects exhibited catalytic activities toward decomposition of P_2_O_5_. These findings were consistent with the results of adsorption state calculations, as covalent interactions and charge transfer between the defects and adsorbed molecules weaken the intramolecular bonds. Having substantiated the possibility of the catalytic dissociation of adsorbed P_2_O_5_ at defect sites, we then calculated the energetics of smaller fragments intercalating through point defects. Phosphorus monoxide (PO), the simplest oxide of phosphorus, was chosen as the subject of this study. Given the positive correlation between an intercalant size and the energy barrier required for its permeation through graphene point defects, we postulate that the intercalation of PO provides a baseline for the minimum energy barrier applicable to the diffusion of any potential phosphorus oxide molecules through monolayer graphene. In the focused reaction coordinate diagram (Figure 3d), we present the energy of three key configurations: the adsorbed state, where the PO molecule adsorbs to the defect site; the transition state, where the molecule penetrates halfway through the defect; and the intercalated state, in which PO has permeated through monolayer graphene but has not yet desorbed or reconfigured itself to the lowest-energy absorption geometry. The energy difference between the transition and adsorbed states indicates the minimum energy barrier required for PO molecules to intercalate monolayer graphene. Similar to P_2_O_5_, PO could not permeate through pristine graphene or Stone–Wales (SW) defects without the introduction of defects of larger sizes. When penetrating through S- and D-defects, the molecule decomposed into individual atoms with the rate limiting step having an energy barrier as high as 8 *eV*. On the other hand, PO was surprisingly permeable at Q-defects. It diffused through the Q-defect without further fragmentation, with a barrier of approximately 2 *eV*, comparable to the energy required to break down chemisorbed P_2_O_5_. Finally, to compare atomic phosphorus and oxygen diffusing through the four different types of defects in graphene (Figure 3e), we performed calculations on (4 × 6) graphene supercells and incorporated results for D- and Q-defects from the work by Song *et. al*.^51^ Remarkably, while point defects enabled both species to intercalate with reduced energy barriers, the energy barrier for individual atoms to penetrate through D- and Q-defects was higher than those for the PO molecule fragments. This observation reiterates the complexity of the interaction process through graphene point defects that cannot be generalized into a single step process.

### Impact of the Intercalants on the Physical Properties of Graphene

**Figure 4:**
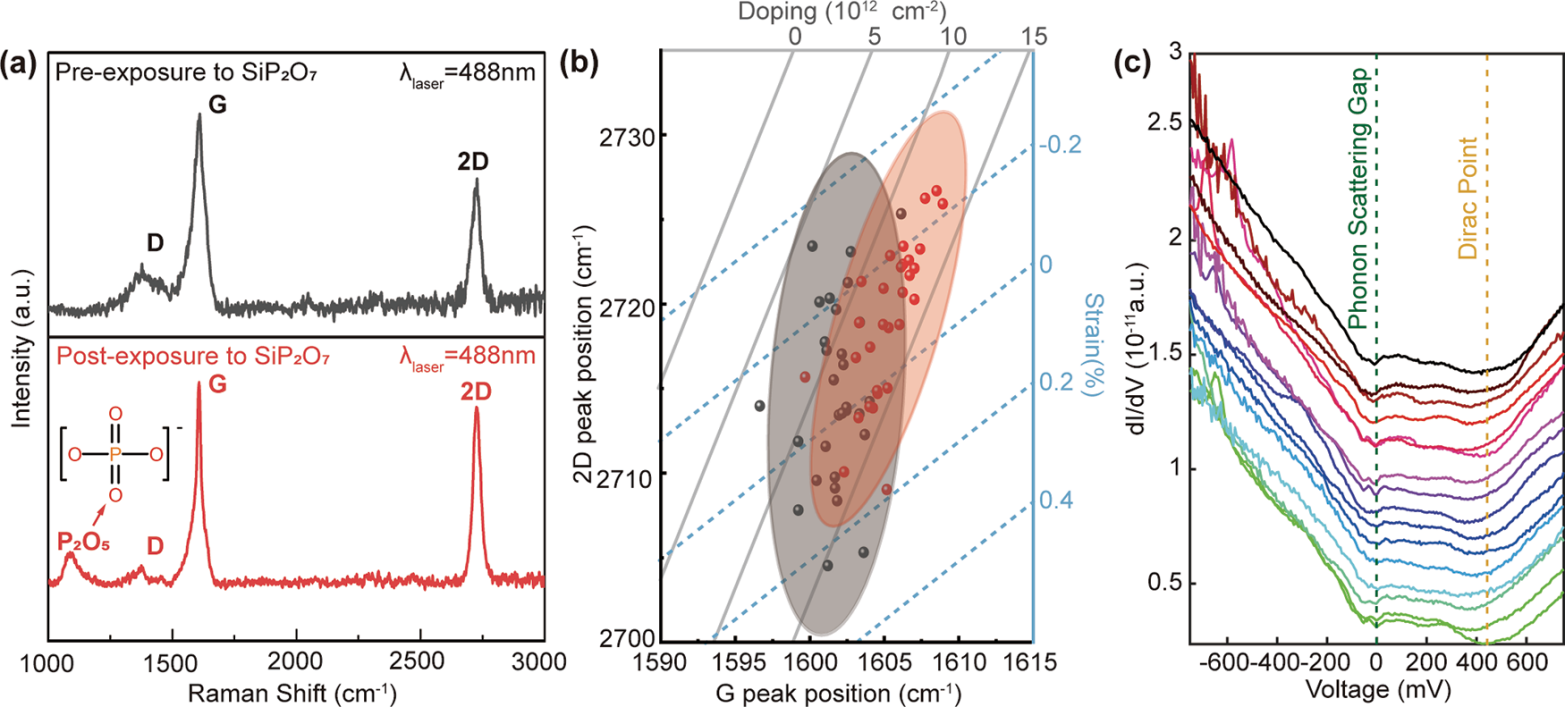
Raman spectra of graphene (a) before (black solid line) and after (red solid line) exposure to an upstream flux of P_2_O_5_. (b) Raman 2D versus G peak position of graphene before (black dots) and after (red dots) exposure to an upstream flux of P_2_O_5_. (c) STS d*I*/d*V* of the intercalated graphene sample. The corresponding phonon scattering gap and Dirac points are highlighted with green dashed line and yellow dashed line, respectively. Spectra are vertically offset for clarity (Tunneling set point = 200 pA, 750 mV).

Graphene inherits highly delocalized π-electrons. Any modification to the spatial extent of the charge density of these π-electrons or tilt of the π-orbitals leads to significant changes to the physical properties of graphene.^52−55^ For example, electronegative or electropositive intercalants confined underneath graphene will lead to charge transfer and doping. This in turn influences the spatial extent of the π-electron charge density and thus the Fermi level of graphene. Moreover, intercalants, such as those confined in graphene bubbles, can also lead to straining of the graphene lattice.^56−58^ This will also change the π-orbital tilt and charge density in graphene, and thus its Fermi level. Therefore, it is expected that the confinement of large P_2_O_5_ molecules at the graphene-germanium heterointerface would lead to significant changes to the physical properties of graphene itself. In our case, we show that charge transfer occurs from graphene to P_2_O_5_, resulting in a p-type doping of the graphene layer. Raman spectroscopy was used to assess changes in the G and 2D peak positions. In Figure 4a, we compare the Raman spectra between nonintercalated and intercalated samples. After intercalation process, both G and 2D peaks blue-shifted after the intercalation process, resulting from changes in strain and doping of graphene due to intercalation.^59,60^ Besides the typical G, 2D and D peaks of graphene, a new Raman peak, approximately at 1100 cm^–1^, was observed (Figure 4a, red solid arrow). This vibrational mode was associated with a  unit in the P_2_O_5_ cage-like structure, and in our case was activated via charge transfer from graphene to the intercalated P_2_O_5_.^61^ The observation of this vibrational mode in Raman, together with the symmetric stretch of bridging oxygen P–O–P bond (Figure S3), further corroborates the formation of the P_2_O_5_ nonplanar cage-like structure at the graphene-substrate heterointerface after intercalation of phosphorus oxide fragments as suggested from our DFT calculations. Furthermore, it is possible to deconvolute the effects of charge transfer and strain on the doping level of graphene by performing a correlation analysis of the G and 2D peak positions between the nonintercalated and intercalated samples (Figure 4b).^62^ In the intercalated samples, a 12% increase in the hole doping level of graphene was observed when compared to the nonintercalated samples. This was further corroborated by scanning tunneling spectroscopy (STS) d*I*/d*V* measurements of the intercalated graphene surface (Figure 4c). The d*I*/d*V* curves were collected on the graphene surface in a line scan with a step of ∼1.1 *nm*. The slope of the d*I*/d*V* curve is proportional to the local density of states (LDOS) of the sample at the tunneling site. Two local minima were observed in all d*I*/d*V* curves, that is, one at *V* = 0 which was the phonon scattering gap (green dashed line), and the another at the Dirac point where the LDOS of graphene reaches its minimum (yellow dashed line).^63^ From Figure 4c, the Dirac point was up-shifted to +425 mV (yellow dashed line) with respect to the Fermi level (*V* = 0) after intercalation, which indicated that P_2_O_5_ led to p-type doping of graphene.

**Figure 5:**
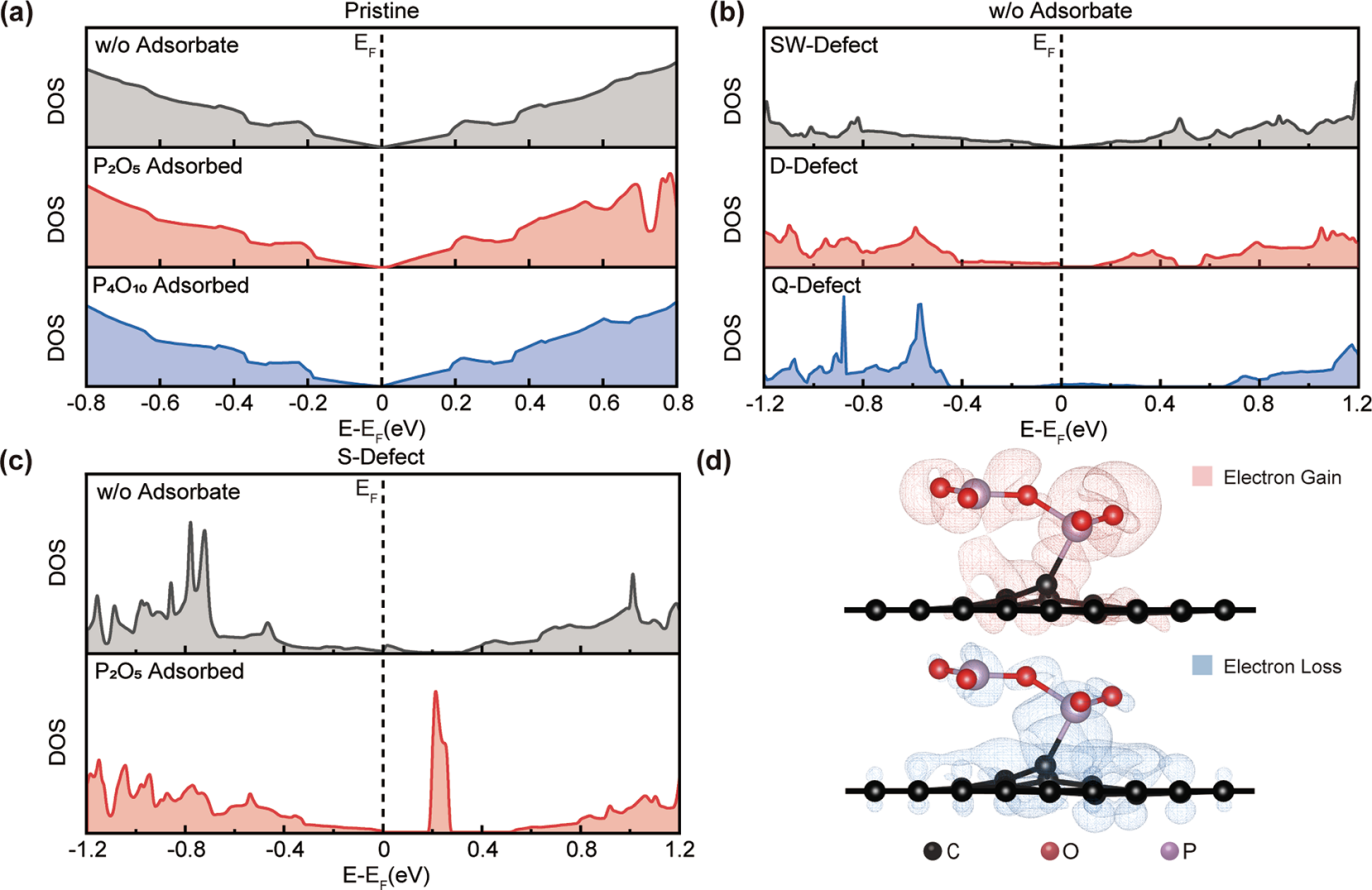
Projected DOS of (a) pristine graphene sheet without (black) and with (red and blue) adsorbed phosphorus oxide, (b) graphene with SW- (black), D- (red), and Q-defects (blue) without adsorbed phosphorus oxide. (c) Comparison of p-orbital DOS of graphene with S-defect (black) and with S-defect covalently bound to P atom in chemisorbed P_2_O_5_ (red). (d) Differential charge density of P_2_O_5_ covalently bound to S-defect. All wireframe isosurfaces denote charge transfer equivalent to 0.001e·Bohr^–3^. Blue isosurface represents charge loss, and red isosurface indicates charge gain.

DFT calculations were also performed to investigate the origins of the observed conductive properties of graphene after intercalation. Given that graphene transferred onto germanium substrates could be subjected to strain,^64−66^ the impact of possible deformation on the electronic structure of monolayer graphene was studied. Our density of states (DOS) calculations based on (4 × 8) supercells of graphene suggested that the electronic structure near the Fermi level did not undergo significant change, despite an increase in the overall system energy with respect to the increased strain. While compressive and tensile stress led to horizontal stretches and compression of the total DOS, respectively, the overall electronic structure near the Fermi level did not vary (Figure S4). We observed that both pristine and deformed graphenes behaved as semimetals. As a result, strain alone did not significantly contribute to the experimentally observed p-type conductive properties in the Raman and STS measurements. While nonuniform strain and formation of bubbles on the graphene monolayer could cause variations in the distance between graphene and underlying germanium substrate, the impact of the germanium surface on the electronic structure of graphene becomes negligible when the separation reaches ∼2 nm (Figure S5). Notably, even in regions where the graphene monolayer was only a few angstroms from the top layer of the germanium surface atoms, the presence of germanium still does not induce semiconducting behavior in graphene. Similarly, while the exposure to P_2_O_5_ caused changes in DOS of graphene far above and below the Fermi level, the overall electronic properties of pristine graphene with adsorbed molecules remained semimetallic, with characteristic converging overlap between the bottom of the conduction band and the peak of valence band (Figure 5a).

Moreover, to elucidate the effect of point defects on altering the bulk conductivity of graphene, the partial DOS of carbon atoms away from the defect sites was also calculated. As shown in Figure 5b, graphene with SW-defects had a conduction band overlapping the valence band at Fermi level, implying semimetallic behaviors as similar to pristine graphene. On the contrary, systems with missing carbon atoms all developed bandgaps and distinct electronic states near the Fermi level. Graphene with S-defects still behaves as a semimetal but develops a gap in its conduction band that is just less than 0.2 eV above the Fermi level. In the case of graphene with D-defects, the bandgap plays an even more noticeable role, as it lies right above the Fermi level, separating the valence band from the conduction band. In systems with Q-defects, the bandgap appears to widen even further, positioning the Fermi level about 0.4 eV above the top of the valence band and 0.7 eV below the bottom of the conduction band. Nevertheless, the enlarged bandgap does not render the system semiconductive. A new, albeit small, state emerges within the band gap and crosses the Fermi level, causing the system to behave more like a metal. Finally, we found that systems whose carbon atoms at defect sites formed covalent bonds to adsorbed P_2_O_5_ fragments underwent substantial changes in their electronic structure due to charge transfer between graphene and adsorbed molecules. DOS of carbon atoms in the defected graphene bond to P_2_O_5_ is illustrated in Figure S6. In all defected systems, the presence of chemisorbed molecules served to enlarge the bandgap and enhance the states in the bandgap, with P_2_O_5_(P) chemisorbed to S-defect being the most notable case (Figure 5c). A summary of these effects is also listed in Table S2. To visualize the loss of electron density in the graphene π-orbitals when P_2_O_5_ was covalently bonded to the graphene defect sites, differential charge calculations were employed (Figure 5d). This change in electron charge density is equivalent to hole-doping of the graphene monolayer, which is consistent with our experimental observations.

### Synthesis of Metal Phosphates at the Heterointerface

**Figure 6:**
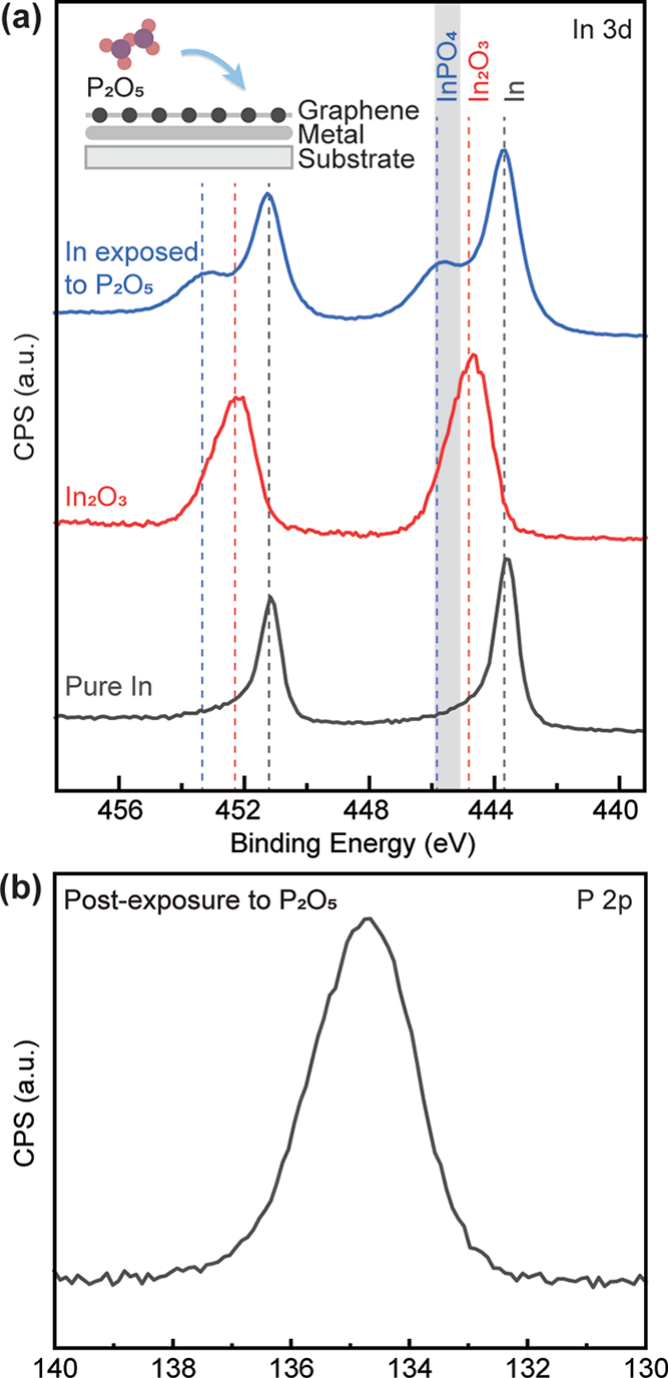
XPS core levels of (a) In 3d for 2D indium confined at the graphene-SiC heterointerface before (black solid line) and after exposure to P_2_O_5_ (blue solid line). The XPS spectra for a pure In_2_O_3_ crystal (red solid line) are also included as a reference. The defined range for InPO_4_ peak positions were evaluated based on data in reference^67,68^ and is highlighted with a shaded gray region. (b) P 2p for the confined 2D indium after exposure to P_2_O_5_.

Although phosphorus has limited solubility^41^ and diffusivity^42^ into germanium, our XPS analysis of the Ge 3d core-level revealed that the intercalation of P_2_O_5_ led to chemical reaction with the germanium substrate. This could have been possible from the intercalated oxygen fragments themselves or the affinity of P_2_O_5_(O) to bond with germanium (Figure 2i). To further elucidate other chemical reactions of the intercalated fragments of P_2_O_5_ that could take place at graphene-substrate heterointerfaces, we show that this process can also be used to form confined metal phosphates by intercalating P_2_O_5_ into a graphene-substrate heterointerface initially containing 2D metals to form indium phosphate as the example in this study (see Methods). Indium phosphate, specifically InPO_4_, is a wide bandgap insulator (*E*_*g*_ = 4.5 eV)^69^ with excellent dielectric properties that initially gain interest as a gate material for InP formed via surface oxidation.^70−73^ However, the formation of dimensionally confined indium phosphates remains largely unexplored, thus impending a more comprehensive understanding of their physical properties and potential applications. In our case, we demonstrated the successful formation of confined indium phosphates by intercalating P_2_O_5_ into a graphene-SiC heterointerface initially containing confined mono- to bilayer indium also formed by intercalation.^17,74^ We identified the composition of the confined indium phosphate at the heterointerface by investigating the In and P core-levels using high-resolution XPS (Figure 6a,b, respectively). In Figure 6a, we compare the core levels of In 3d, before (black solid line) and after (blue solid line) intercalation of P_2_O_5_ to the graphene-substrate heterointerface containing 2D indium, to the In 3d core level of a pure In_2_O_3_ reference sample (red solid line). In addition to the typical peaks for indium metal (black dashed line), the XPS spectra exhibited new peaks around 445.8 and 453.3 eV in the intercalated sample (blue dashed line). These two new peaks were up-shifted by 1.1 eV from the peak position of the In_2_O_3_ reference (red dashed line).^75^ Furthermore, a strong phosphorus signal was detected in samples intercalated with P_2_O_5_ (Figure 6b). Therefore, based on these observations, we can exclude the formation of In_2_O_3_ in the P_2_O_5_ intercalated samples. Further inspection and comparison of our XPS core-level spectra to previous reported XPS of indium phosphates suggest that the peaks at 445.8 and 453.3 eV are the In 3d core-levels associated with P–O–In 3d_3/2_ and P–O–In 3d_5/2_ bonding, respectively. Although the phase of the confined indium phosphate at the graphene-substrate heterointerface was likely in the form of InPO_4_, In(PO_3_)_3_ and other metastable oxide phases could also form at such confined spaces^38,67,68^ and therefore are important to investigate in future studies.

## Conclusions

In summary, we demonstrated that large molecules, such as P_2_O_5_, can permeate the basal plane of graphene and intercalate into the heterointerface. This was evident from the strong P and O signals that were detected underneath the graphene surface from spectroscopic measurements (EDS, nano-FTIR, XPS, Raman, and STS) and microscopic cross-sectional imaging and analysis in TEM. Although the physical size of the molecule was larger than that of the graphene lattice, fragments of the molecule could intercalate through typical pathways in the graphene lattice. This was revealed by calculating the penetration barriers for fragments of P_2_O_5_ of varied size and chemistry through pristine and defected graphene of different configurations. The complexity of the interaction process of large molecules through the basal plane of graphene cannot be generalized into a single step process. However, a two-step mechanism, based on realistic permeation energy barriers, was proposed for the intercalation of P_2_O_5_: (i) P_2_O_5_ first dissociated into small fragments (i.e., P, O, etc.) catalyzed by point defects in the graphene lattice; (ii) these fragments once intercalated can chemically react at the confined graphene–substrate heterointerface forming a condensed phase that chemically resembles the initial state of the molecule. It has been shown that the intercalation of P_2_O_5_ at the graphene-substrate heterointerface could effectively tune the doping level of graphene via charge transfer. Moreover, the intercalated P_2_O_5_ (and/or its fragments) can also act as reactants and further contribute to other interfacial reactions, for example, the conversion of 2D indium into 2D indium phosphate at the heterointerface between graphene and SiC. Although our findings only focus on indium phosphates, our results demonstrate the potential of this approach for the conversion of other metals (e.g., Ga, Al, etc.) and alloys. Furthermore, spatial control over the distribution of defects, such as localized irradiation using plasma processing,^76^ can promote the selective area intercalation of metals and/or alloys under specific regions of graphene. While the focus of this study is on P_2_O_5_ intercalation, the possibility of intercalation from predissociated molecules catalyzed by defects in graphene may exist for other types of molecules as well. This in-depth study advances the comprehension of intercalation routes of large molecules via the basal plane of graphene as well as heterointerface chemical reactions leading to the formation of distinctive confined complex oxide compounds.

## Methods

### Intercalation of P_2_O_5_ at Graphene–Substrate Heterointerfaces

The intercalation process was performed at 1 atm in a single-zone furnace under argon flow. Two kinds of samples were used in this study: transferred CVD graphene (Graphenea) onto cleaned Ge(110) substrates (MTI Corp.) and 2D indium samples intercalated at graphene-SiC(0001) heterointerface, as reported by Rajabpour et al.^17^ SiP_2_O_7_ (Saint-Gobain Inc.) was utilized to produce an upstream flux of P_2_O_5_ to the samples. First, the center of the furnace was set to 950 °C at a ramping rate of 55 °C/min (stage I) and then dwelled at 950 °C for 45 min (stage II). In stage II, the measured temperature for the sample and SiP_2_O_7_ was 600 and 950 °C, respectively. Samples were then naturally cooled to room temperature under argon flow.

### Materials Characterization

SEM and EDS were performed on a Thermo Fisher Scios 2 dual-beam microscope. The cross-sectional samples of graphene bubbles for TEM analysis were prepared using a focused ion beam (FIB) Zeiss NVision 40 FIB-SEM. The target location was extracted from the specimen and attached to a copper FIB grid using a conventional FIB milling and lift-out procedure. The HAADF image and STEM-EDS mappings on the cross-section of graphene bubbles were collected with a Thermo Fisher Scientific Talos 200× operated at 200 kV. HAADF was performed with a spot size less than 1 nm with a convergence semiangle of 10.5 mrad. Furthermore, XPS spectra and depth profiles were carried out in a Thermo-Fisher K-Alpha Plus XPS system and in a Kratos Axis Ultra DLD system using a monochromatic Al Kα source (*h*ν = 1486.6 eV). Raman measurements were performed on a Renishaw inVia system with a 488 nm laser (maximum power at 100 mW). STM was performed in a closed-cycle RHK Pan Scan Freedom SPM system in UHV (∼1 × 10^–10^ mbar) at a base temperature of 10 K. d*I*/d*V* measurements were taken using a built-in digital lock-in amplifier operating at 1.3156 kHz with a 10 mV excitation amplitude. Moreover, nano-FTIR of the graphene bubbles was performed on a commercial scattering-type scanning near-field optical microscope (s-SNOM) setup (Neaspec Inc.), and the collected spectra were normalized to that obtained on a silicon sample using the same acquisition parameters. Nano-FTIR of the P_2_O_5_ reference sample was performed on a synchrotron infrared light-based nano-spectroscopy (SINS) setup (Innova, Bruker) (beamline 5.4, Advanced Light Source, Lawrence Berkeley National Laboratory).

### First-Principles Calculation

DFT calculations were performed using the generalized gradient approximation (GGA) with the Vienna ab initio simulation package. The projector-augmented-wave (PAW) method was used, and Grimme’s DFT+D2 was applied to better account for the van der Waal’s interaction between graphene monolayer and absorbed species.^77^ The kinetic energy cutoff was set at 500 eV, and the global convergence criterion for breaking electronic SC-loop was chosen to be 5 × 10^–5^. A vacuum space of 22 Å was applied on top of the graphene sheet to best model the isolated state of a monolayer. Γ point sampling was used throughout all calculations. For relaxations and nudged elastic band (NEB) calculations, a grid of 5 × 4 × 1 was used. To obtain density of states (DOS), a grid of 9 × 9 × 1 was adopted for best precision. For more information on simulating the reaction mechanism, see the Supporting Information.

## Data Availability

All data needed to evaluate the conclusions in the paper are present in the manuscript and/or the Supporting Information.

## Supporting Information Available

Additional information including details of graphene transfer process and first-principles calculation, intercalation of P_2_O_5_ molecule through a defect of 9 vacant atoms, XPS depth profile for graphene/Ge (110) after exposure to P_2_O_5_, projected DOS of carbon atoms in deformed graphene sheet, projected DOS of carbon in defected graphene with P_2_O_5_ adsorbed to atoms at defect site (PDF)
