# Supplementary material for: Elucidating the Mechanism of Large Phosphate Molecule Intercalation Through Graphene-Substrate Heterointerfaces
Source: ACS Appl Mater Interfaces. 2023 Oct 2;15(40):47649–60. doi: 10.1021/acsami.3c07763 (PMC10571006; doi:10.1021/acsami.3c07763)
Supplement: Supplementary file 1 — am3c07763_si_001.pdf [file am3c07763_si_001.pdf]

Supporting Information

Elucidating The Mechanism of Large Phosphate Molecule Intercalation Through Graphene-Substrate Heterointerfaces

Jiayun Liang[1], Ke Ma[1], Xiao Zhao[1,2], Guanyu Lu[3], Jake Riffle[4], Carmen M. Andrei[5], Chengye Dong[6], Turker Furkan[7], Siavash Rajabpour[7], Rajiv Ramanujam Prabhakar[8], Joshua A. Robinson[6,7], Magdaleno Jr. Vasquez[10], Quang Thang Trinh[11], Joel W. Ager[1,2], Miquel Salmeron[1,2], Shaul Aloni[9], Joshua D. Caldwell[3], Shawna Hollen[4], Hans A. Bechtel[12], Nabil D. Bassim[5,13], Matthew P. Sherburne[1], Zakaria Y. Al Balushi[1,2,*]

1. Department of Materials Science and Engineering, University of California, Berkeley, Berkeley, CA 94720, USA.
2. Materials Sciences Division, Lawrence Berkeley National Laboratory, Berkeley, CA 94720, USA.
3. Department of Mechanical Engineering, Vanderbilt University, Nashville, TN 37235, USA.
4. Department of Physics and Astronomy, University of New Hampshire, Durham, NH 03824, USA.
5. Canadian Centre for Electron Microscopy, McMaster University, Hamilton ON L8S 4L8, Canada.
6. 2D Crystal Consortium, The Pennsylvania State University, University Park, PA 16802, USA.
7. Department of Materials Science and Engineering, The Pennsylvania State University, University Park, PA 16802, USA.
8. Chemical Sciences Division, Lawrence Berkeley National Laboratory, Berkeley, CA 94720, USA.
9. The Molecular Foundry, Lawrence Berkeley National Laboratory, Berkeley, CA 94720, USA.
10. Department of Mining, Metallurgy, and Materials Engineering, University of the Philippines, Diliman, Quezon City 1101, Philippines
11. Queensland Micro- and Nanotechnology Centre, Griffith University, Brisbane, 4111 Australia
12. Advanced Light Source, Lawrence Berkeley National Laboratory, Berkeley, CA 94720, USA.
13. Department of Materials Science and Engineering, McMaster University, Hamilton ON L8S 4L8, Canada.

[*]To whom correspondence should be addressed. e-mail: albalushi@berkeley.edu

This PDF file includes:

Supporting Text

- Graphene Transfer Process
- Details on First Principles Calculation
- Intrinsic Defects in Monolayer Graphene

Supporting Tables

- Table S1. Summary of absorption mechanisms of P_2O_5 and P_4O_{10} on pristine and defected graphene monolayer.
- Table S2. Conductive properties of pristine and defected graphene sheets exposed to P_2O_5 molecules.

Supporting Figures

- Figure S1. XPS depth profiles of graphene-germanium intercalated with P_2O_5.Intercalation of P_2O_5 through a defect of 9 vacant atoms.
- Figure S2. Intercalation of P_2O_5 through a defect of 9 vacant atoms.
- Figure S3. Raman spectra of the post-intercalated sample in lower frequency range.
- Figure S4. Projected DOS of carbon atoms in deformed graphene sheet.
- Figure S5. Projected DOS of carbon atoms in graphene sheet separated from underlying germanium substrate.
- Figure S6. Projected DOS of carbon in defected graphene with P_2O_5 adsorbed to atoms at defect site.

SUPPORTING TEXT

Graphene Transfer Process

1cm by 1cm CVD-grown monolayer graphene (MLG) on polymer film (Graphenea) was placed into deionized water slowly and detached from the supporting polymer film underneath. Afterwards, a clean Ge (110) substrate was then introduced into deionized water and fished the graphene from below, followed by 30-mins dry in air and another 1-hour heating treatment at 150 °C on a hotplate. Later, graphene-Ge (110) sample was kept in low pressure environment (~10^{-3} Torr) overnight and annealed at 450 °C in N_2 atmosphere for 2 hours.

Details on First Principles Calculation

When simulating the dissociation of phosphorous oxide molecules and hetero atom intercalation through defected graphene monolayer, climbing image nudged elastic band (CI-NEB) calculations were performed to determine the most probable reaction route[1]. Such process involves three steps. First, the most stable initial and final configurations were obtained by structural relaxation. Then a CI-NEB calculation with 6-8 intermediates was conducted to find a most realistic energy path connecting the two configurations. Lastly, the two intermediates closest to the maxima on energy profile were used as initial and final configurations for another NEB calculation of 3-4 images to precisely determine the geometry and energy profile of intermediate states. To accurately model P_2O_5 dissociation reactions of complex potential energy surface, multiple plausible chains of images were generated, and NEB calculations were performed for each. The energy profile of all chains was examined and the pathway exhibiting the lowest energy barrier was identified as the most probable route for the reaction.

Intrinsic Defects in Monolayer Graphene

Four types of intrinsic defects of graphene were studied via DFT. Stone-Wales defects (denoted as SW-Defects in **Fig. 4a**) are created by rotating a single pair of carbon atoms by 90 degrees to form adjacent pairs of pentagonal and heptagonal rings. The formation of Stone-Wales defects does not involve the introduction or removal of any atoms. Single vacancy defect (denoted as S-Defect in **Fig. 4a**) is formed when a single carbon atom is removed from a hexagon ring in graphene monolayer. In response to the removal of single atom, the structure near the vacancy undergoes Jahn-Teller distortion to minimize total energy, leading to a decreased separation between carbon atoms near the vacancy. Similarly, double vacancy defects (D-Defects in **Fig. 4a**) are formed through the loss of two adjacent carbon atoms in pristine monolayer graphene. Lastly, quadruple vacancy defects (Q-Defects in **Fig. 4a**) are produced by removing one carbon atom and its three nearest neighbors from the hexagon, resulting in a total of four missing atoms.

SUPPORTING REFERENCES

1. Henkelman, G.; Uberuaga, B. P.; Jónsson, H. A Climbing Image Nudged Elastic Band Method for Finding Saddle Points and Minimum Energy Paths. *The Journal of chemical physics* **2000,** *113*, 9901-9904.

SUPPORTING TABLES

Table S1. Summary of absorption mechanisms of P_2O_5 and P_4O_{10} on pristine and defected graphene monolayer. The notation (P) denotes that the molecule is binding or approaching graphene primarily through the phosphorous atom, while (O) represents adsorption primarily through the oxygen atom.

Adsorption Mechanism	P_2O_5(P)	P_2O_5(O)	P_4O_{10}(P)	P_4O_{10}(O)
Pristine Graphene	vdW	vdW	vdW	vdW
SW-Defect	Covalent	Reaction	vdW	vdW
S-Defect	Covalent	Covalent	vdW	vdW
D-Defect	Covalent	vdW	vdW	vdW
Q-Defect	Covalent	Reaction	vdW	vdW

Table S2. Conductive properties of pristine and defected graphene sheets exposed to P_2O_5 molecules.

	Without Absorbate	P_2O_5(P)	P_2O_5(O)
Pristine Graphene	Semimetal	Semimetal	Semimetal
SW-Defect	Semimetal	Semimetal	Semimetal
S-Defect	Semimetal	P-type	P-type
D-Defect	P-type	Semimetal	P-type
Q- Defect	Metal	Metal	Metal

SUPPORTING FIGURES

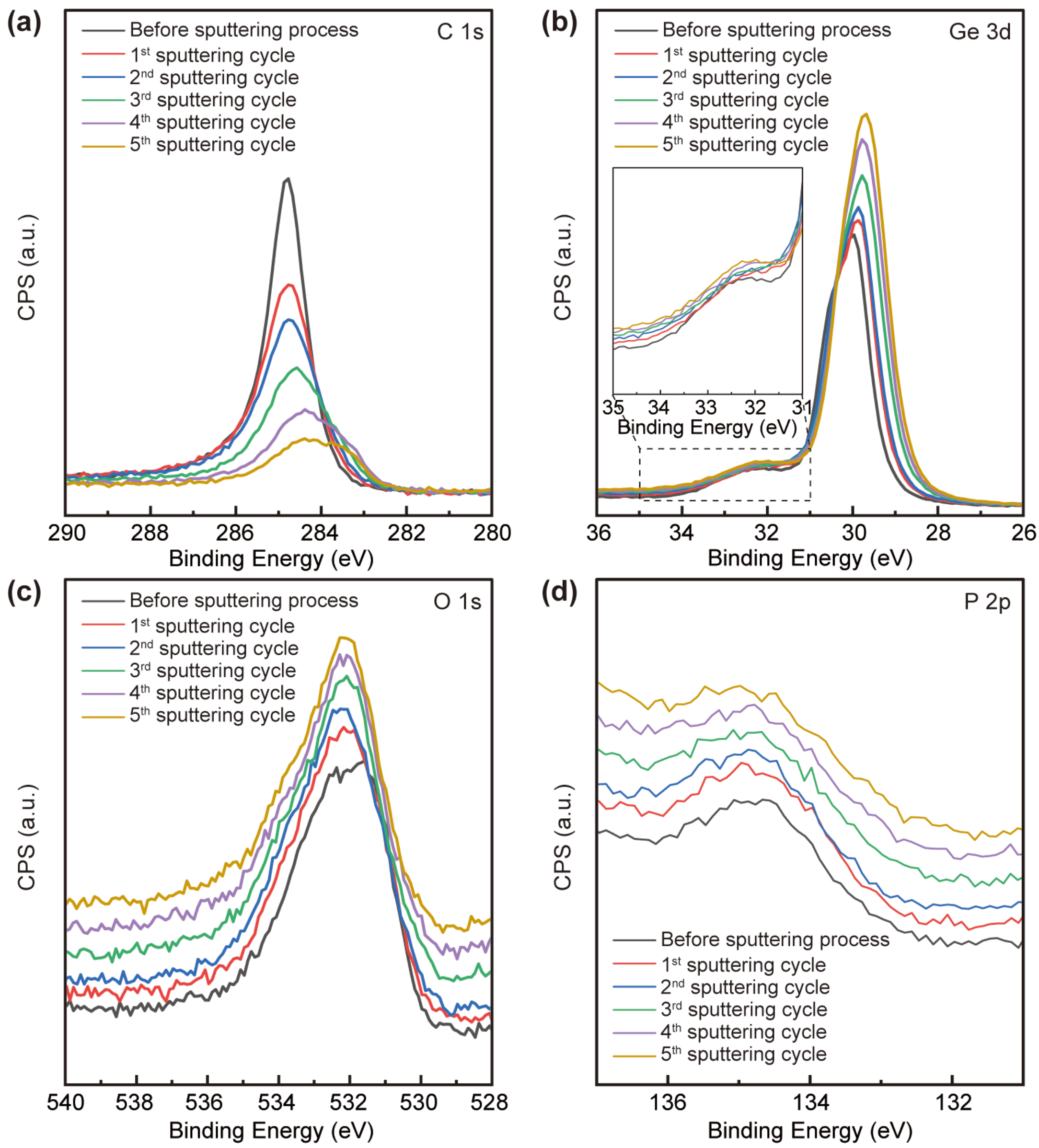

Figure S1. XPS depth profile of graphene-germanium after exposure to P_2O_5. High-resolution XPS spectra collected before the ion sputtering process (*black*); after the first sputtering cycle (*red*); after the second sputtering cycle (*blue*); after the third sputtering cycle (*green*); after the fourth sputtering cycle (*violet*) and after the fifth sputtering cycle (*yellow*) for core-levels of (a) C 1s, (b) Ge 3d, (c) O 1s, and (d) P 2p. In the XPS spectra of Ge 3d, region where the binding energy ranged from 31 eV to 35 eV was magnified to highlight the intensity change of the germanium oxide.

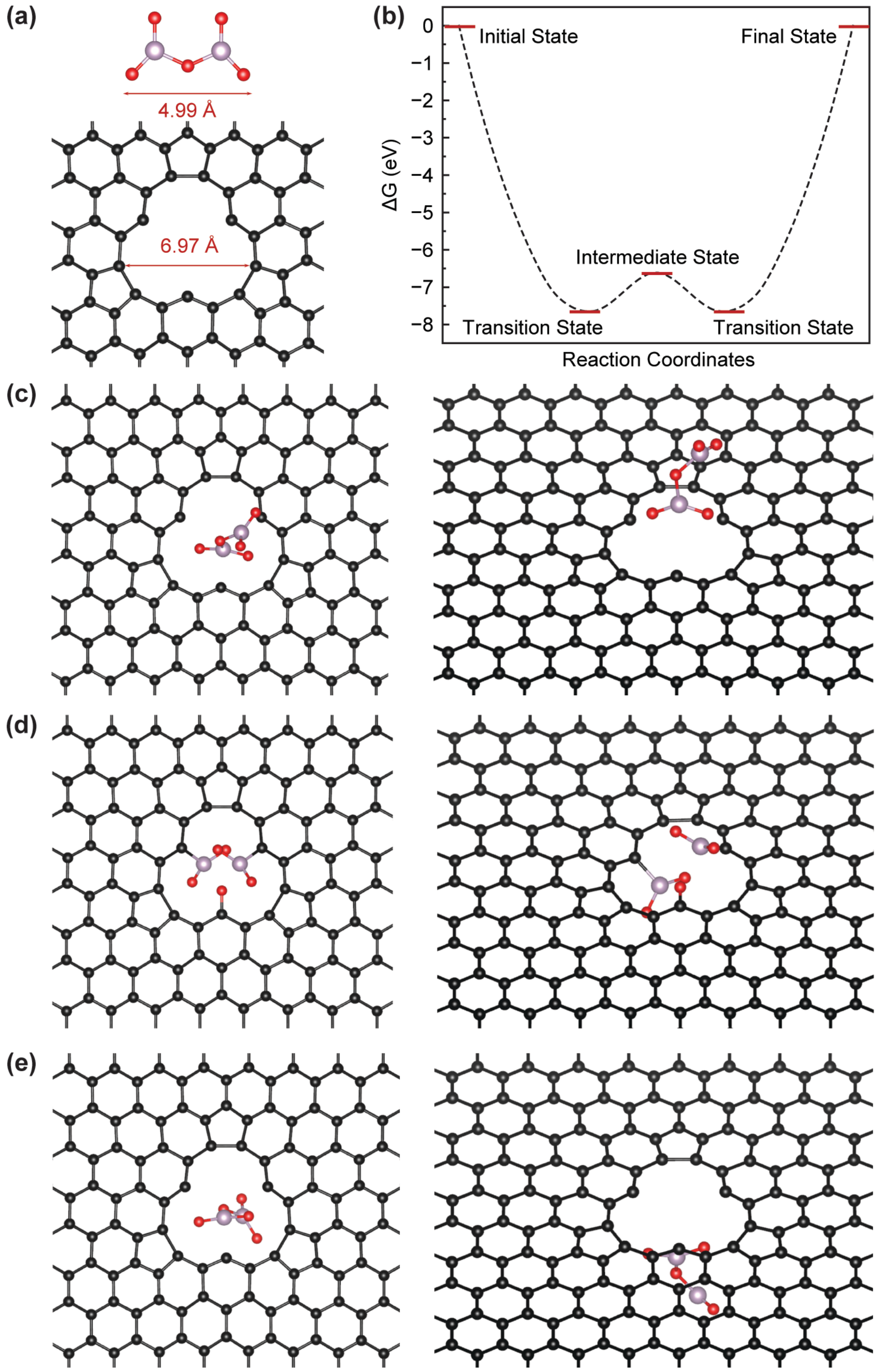

Figure S2. Intercalation of P_2O_5 molecule through a defect of 9 vacant atoms. (a) A comparison of dimensions of a defect of 9 vacant atoms and P_2O_5 molecule. (b) Energy profile of P_2O_5 intercalation through a 9-vacant-atom defect. The molecule undergoes spontaneous dissociation upon contact with the defect site and would require an energy barrier of 7.7eV to desorb and recombine on the opposite side of the graphene monolayer. Top (*left*) and angled (*right*) view of the (c) Initial, (d) Intermediate, and (e) Final state configuration of P_2O_5 molecule during intercalation.

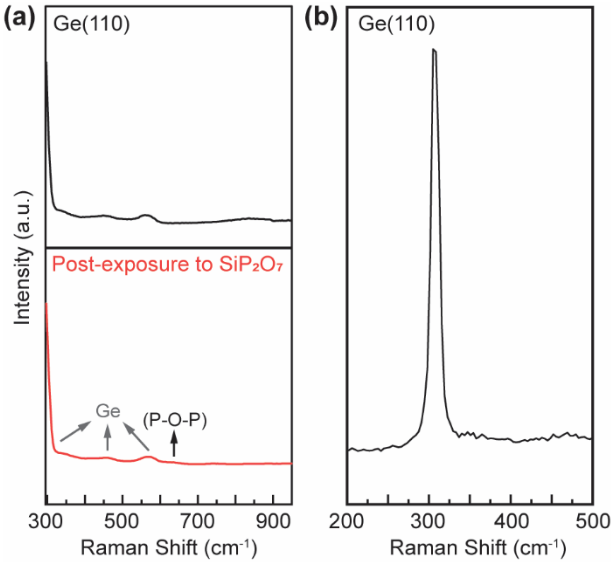

Figure S3. Raman spectra of the post-intercalated sample in lower frequency range. (a) Ge (110) substrate without exposure to SiP_2O_7 *(black solid line) and* after exposure to SiP_2O_7 *(red solid line)* from 300 to 950 cm^{-1}. (b) Raman spectra of Ge (110) substrate without exposure to SiP_2O_7 from 200 to 500 cm^{-1}.

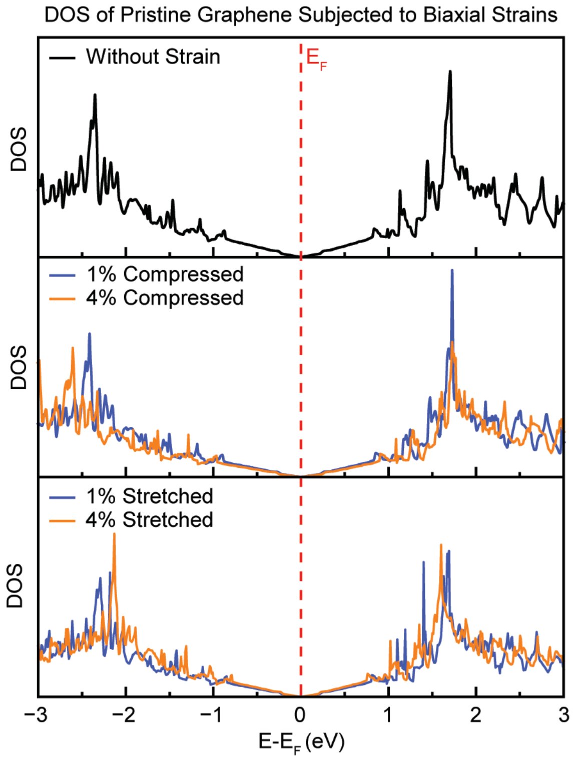

Figure S4. Projected DOS of carbon atoms in deformed graphene monolayer.

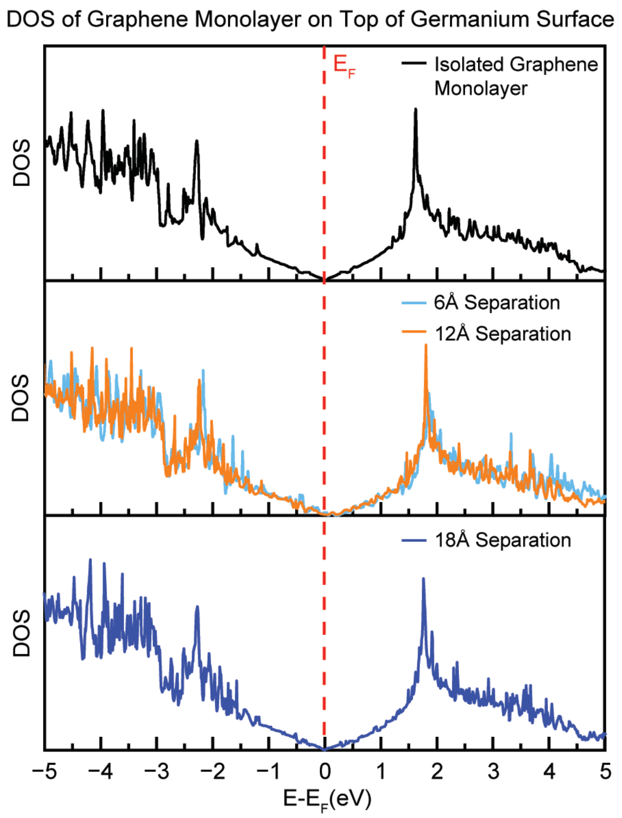

Figure S5. Projected DOS of carbon atoms. The top panel shows the DOS for carbon atoms in an isolated, free-standing graphene monolayer. The middle and bottom panels present the DOS for carbon atoms in graphene sheet separated from the underlying germanium surface at various distances.

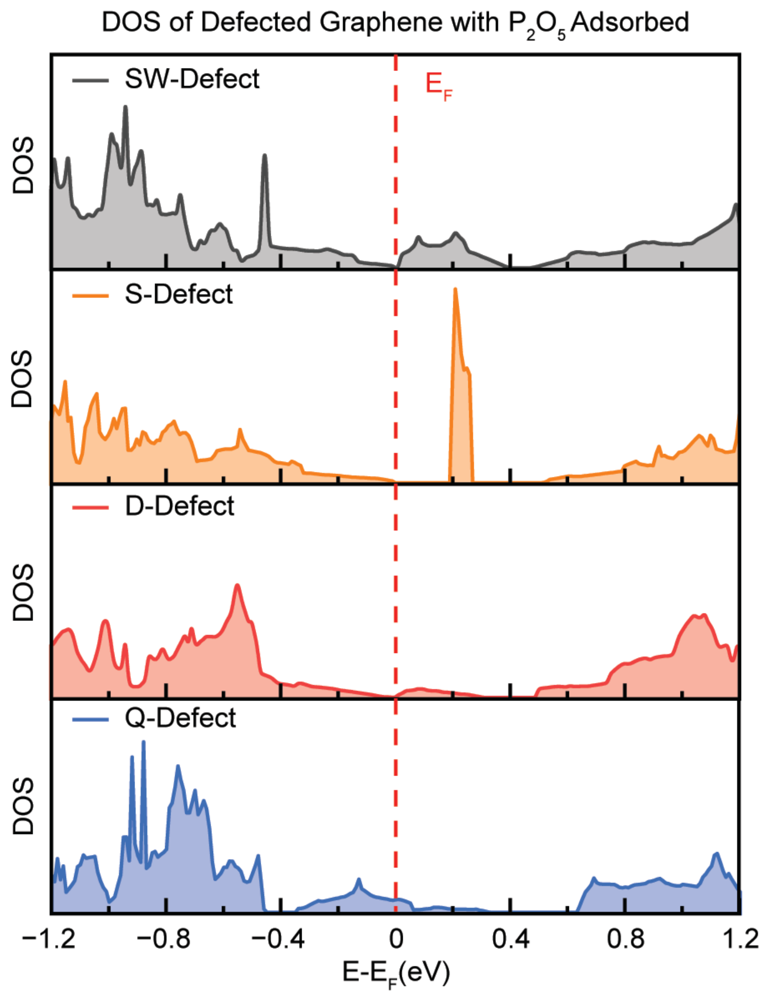

Figure S6. Projected DOS of carbon in defected graphene monolayer with P_2O_5 adsorbed to atoms at defect site.
